# Severe Abdominal Pain as a Prominent Clinical Manifestation of Anti‐DPPX Autoimmune Encephalitis: A Case Report and Systematic Review

**DOI:** 10.1002/iid3.70472

**Published:** 2026-06-11

**Authors:** Difang Shi, Haohao Wu, Baogang Huang, Yan Zheng, Jia Liu, Jianjian Bao, Fengming Xu, Kang Du

**Affiliations:** ^1^ Department of Thoracic Surgery I, The Third Affiliated Hospital of Kunming Medical University, Yunnan Cancer Hospital Peking University Cancer Hospital Yunnan Kunming Yunnan China; ^2^ Department of Neurology Yunnan Qujing Central Hospital (Qujing First People's Hospital) Qujing Yunnan China

**Keywords:** autoimmune encephalitis, case report, DPPX, literature review, severe abdominal pain

## Abstract

**Background:**

Anti‐dipeptidyl‐peptidase‐like protein 6 encephalitis (DPPXE) is an exceptionally rare form of autoimmune encephalitis characterized by a highly heterogeneous clinical phenotype.

**Methods:**

In this study, we report a Chinese patient presenting with severe abdominal pain as a prominent symptom; furthermore, we conducted a systematic review and analysis of 125 cases of DPPXE (including the present case).

**Results:**

This rare case presented with severe abdominal pain as a prominent symptom. We comprehensively summarized the clinical presentations, patterns of recurrence, and therapeutic responses of DPPXE. Notably, by comparing the clinical characteristics of DPPXE cases reported in China with those from other regions, we found some differences in disease presentation, suggesting potential racial or geographic variability.

**Conclusion:**

Our findings may provide up‐to‐date and comprehensive insights into the pathophysiology and clinical course of this rare disorder from multiple perspectives.

## 1 Introduction

Autoimmune encephalitis represents a group of inflammatory disorders of the central nervous system (CNS), characterized by the presence of autoantibodies targeting neuronal surface antigens, ion channels, or receptors [1]. Among these, anti‐dipeptidyl‐peptidase‐like protein 6 encephalitis (DPPX encephalitis, DPPXE) represents a rare subtype, first described by Boronat et al. in 2013 [2]. DPPX, a critical co‐modulatory subunit of voltage‐gated α‐type Kv4.2 channels, is abundantly expressed in the neuronal cytosol and dendrites within the hippocampus, cerebellum, and enteric nervous system. It functions to enhance transient outward Kv4.2‐mediated potassium currents, thereby modulating the backpropagation of action potentials in neurons [2, 3, 4, 5]. However, the generation of anti‐DPPX antibodies disrupts this regulatory mechanism, resulting in heightened neuronal excitability, which clinically manifests as a characteristic triptych of weight loss, CNS hyperexcitability, and cognitive impairment [6]. With an increasing number of reported cases, it has become evident that the clinical phenotype of DPPXE is highly heterogeneous, beyond the classical triptych; symptoms such as gastrointestinal dysfunction, abdominal pain, headache, limb pain, sleep disturbances, and ataxia have been documented, indicating a broader clinical spectrum [2, 6, 7, 8, 9, 10, 11, 12, 13, 14]. Notably, abdominal pain has been reported in eight cases in the literature, but the pain was generally mild, transient, and not considered a predominant clinical feature [2, 10, 14, 15, 16]. Among these, severe abdominal pain has rarely been described, and only one previously reported case explicitly identified it as a core manifestation of DPPXE [10]. In this study, we present an additional rare case of DPPXE accompanied by epilepsy and severe epigastric pain as a prominent symptom. Additionally, we systematically review the existing literature to comprehensively summarize the clinical characteristics of this disease, thereby enhancing awareness, facilitating diagnosis, and promoting early intervention of this rare encephalitis subtype.

## 2 Case Presentation

An 18‐year‐old male was admitted with intermittent episodic limb convulsions accompanied by severe abdominal pain persisting for over 3 years, with recurrence of severe abdominal pain for 5 days before admission to our hospital. The patient's symptoms initially manifested more than 3 years ago as episodic limb convulsions without identifiable triggers, accompanied by loss of consciousness, intense epigastric abdominal pain, profuse diaphoresis, each episode lasted approximately 5 min and resolved spontaneously, followed by postictal weakness without hemiparesis, anxiety, chest pain, or other neurological deficits. The patient was diagnosed with epilepsy at a local hospital and was treated with oral sodium valproate as antiepileptic therapy, in addition to analgesic and gastroprotective agents, including diclofenac sodium, omeprazole, sucralfate, and resorcinol, resulting in only modest symptomatic relief. During the subsequent course, the patient had recurrent seizures and severe abdominal pain of similar intensity. The abdominal pain occurred in two patterns: (1) temporally associated with seizures; (2) as isolated episodes of sudden severe epigastric pain without accompanying epileptic features, with episode duration of 5–30 min in both patterns, with the latter pattern being predominant. Over the past year, the frequency of both seizures and severe abdominal pain increased compared to earlier periods, with abdominal pain becoming particularly frequent. He sought treatment at multiple hospitals and was administered antiepileptic therapy with levetiracetam (1.0 g, Q12h), lacosamide (100 mg, bid), phenytoin sodium (100 mg, bid), and clonazepam (0.25 mg, bid). Analgesics were also concurrently administered to manage abdominal pain; although seizure frequency decreased, the severe abdominal pain persisted during episodes without significant improvement.

Five days prior to admission, the patient developed recurrent severe upper abdominal pain of unclear origin. During this hospitalization at our hospital, episodes were predominantly isolated abdominal pain, with only one episode occurring in temporal association with a seizure. The pain intensity reached 9 on a numeric rating scale, with episodes accompanied by profuse diaphoresis and significant functional impairment requiring bed rest. Antispasmodic and analgesic therapy with resorcinol proved ineffective, intramuscular administration of pethidine hydrochloride resulted in partial pain relief; subsequent treatment with continuous intravenous infusion of midazolam, along with oral administration of levetiracetam and lacosamide, led to a reduction in seizure frequency. However, both seizures and severe abdominal pain continued to recur intermittently during hospitalization. He had a history of hepatic insufficiency that had not undergone systematic evaluation or treatment. About a year ago, a thyroid nodule was identified during a routine physical examination, but no further diagnostic workup was performed. There was no history of toxic exposure, parasitic infection, or other relevant environmental factors. Both his parents were in good health, with no known family history of hereditary or genetic disorders.

Neurological physical examination revealed a body temperature of 36.6°C, heart rate of 75 beats per minute, respiratory rate of 19 breaths per minute, blood pressure of 101/80 mmHg, and an oxygen saturation of 94%. The patient was in poor general condition and presented with an altered mental status; bowel and bladder function remained normal. Examination of higher cortical functions and cranial nerves revealed no abnormalities. Sensory function was intact bilaterally, and muscle strength was grade 5 in all four limbs, with normal muscle tone. Deep tendon reflexes were normally elicitable, and bilateral coordination tests demonstrated no abnormalities. However, bilateral Kernig's sign was positive, accompanied by nuchal rigidity. Physical examination of the digestive system revealed a flat and symmetrical abdomen, without muscular guarding, no tenderness, rebound tenderness, or palpable abdominal masses were detected. Murphy's sign and McBurney's point tenderness were negative.

In auxiliary examinations, his complete blood count was within normal limits, biochemical analysis indicated hepatic dysfunction, characterized by elevated alanine aminotransferase (324 U/L) and aspartate aminotransferase (158 U/L) levels, hyperglycemia, and impaired coagulation parameters. Thyroid function tests and electrolyte levels were within normal limits. Serological assays for human immunodeficiency virus antibodies, treponema pallidum antibodies, hepatitis C virus antibodies, hepatitis B virus antigen and antibody profiles, as well as tumor markers, showed no abnormalities. Computed tomography (CT) of the chest and abdomen (non‐contrast and contrast‐enhanced) revealed hepatic steatosis and multiple small pulmonary nodules, with no evidence of underlying malignancy. Subsequent gastrointestinal endoscopy with histopathological evaluation demonstrated chronic colitis without features indicative of ulcerative colitis, Crohn's disease, ulcerations, or gastrointestinal neoplasms. His brain MRI showed no significant abnormalities. During 48‐h video electroencephalography (EEG) monitoring, two typical episodes of isolated abdominal pain without motor manifestations were captured, with no ictal epileptiform discharges or evolution; overall findings were unremarkable.

**Figure 1:**
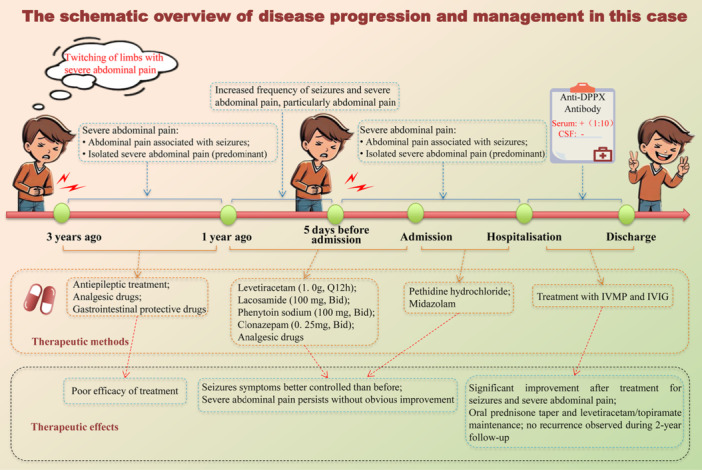
The schematic overview of disease progression and management in this case. Created using Adobe Illustrator.

During hospitalization, the patient continued to experience recurrent convulsive seizures accompanied by severe abdominal pain. Given the clinical course and symptomatology, autoimmune encephalitis was suspected. Serum and cerebrospinal fluid (CSF) testing for autoimmune encephalitis‐related antibodies revealed serum anti‐DPPX positivity (titer 1:10), but negativity in CSF, and CSF analysis showed a white blood cell count of 4/μL, protein level of 36 mg/dL, and negative oligoclonal bands. Repeat testing of serum 1 day later confirmed persistent serum positivity at the same titer. Based on the above findings, the definitive diagnosis of anti‐DPPX antibody‐associated autoimmune encephalitis was established. The patient subsequently received high‐dose intravenous methylprednisolone (IVMP; 1000 mg/day) as pulse therapy, in combination with intravenous immunoglobulin (IVIG). Concurrent oral antiepileptic therapy with levetiracetam and lacosamide was continued. Following treatment, the patient's abdominal pain and seizure frequency showed significant improvement, and he was discharged in a clinically stable condition with no recurrence observed at the time of discharge under this therapeutic approach. Following discharge, the patient was transitioned to oral prednisone at 1 mg/kg/day for 2 weeks, followed by a gradual taper of 5 mg every 2 weeks over a total course of approximately 6 months, and continued maintenance antiepileptic therapy with levetiracetam (1 g every 12 h) in combination with topiramate (25 mg twice daily). At the 2‐year follow‐up, no recurrence of seizures or severe abdominal pain was reported, and the modified Rankin Scale score was 0. The longitudinal clinical history of the patient, including diagnostic timelines and treatment responses, is outlined in Figure 1.

## 3 Methods of Literature Review

### 3.1 Data Collection and Eligibility Criteria

A comprehensive literature search was conducted using the keywords “anti‐DPPX encephalitis,” “DPPX antibody,” “anti‐DPPX autoimmune encephalitis,” “DPPX encephalitis,” and “dipeptidyl peptidase‐like protein‐6” in both English and Chinese databases, employing the Boolean operator “OR.” The search spanned from January 2013 to November 2025 across PubMed, Web of Science, Embase, Cochrane Library, China National Knowledge Infrastructure (CNKI), and Wanfang databases to identify clinical studies and case reports related to anti‐DPPX antibody encephalitis. Inclusion criteria were defined as follows: (1) studies reporting individual or series of cases of anti‐DPPX antibody encephalitis with confirmed diagnosis; (2) availability of detailed clinical, radiological, or laboratory data. Exclusion criteria comprised duplicate records, publications in languages other than English or Chinese, non‐human studies, studies unrelated to anti‐DPPX encephalitis, reports with incomplete case details, secondary literature such as reviews, meta‐analyses, commentaries, or letters lacking sufficient patient information, and cases with unclear diagnosis or insufficient clinical evidence. All eligible studies were independently assessed by two investigators, and any disagreements were resolved by a third expert.

**Figure 2:**
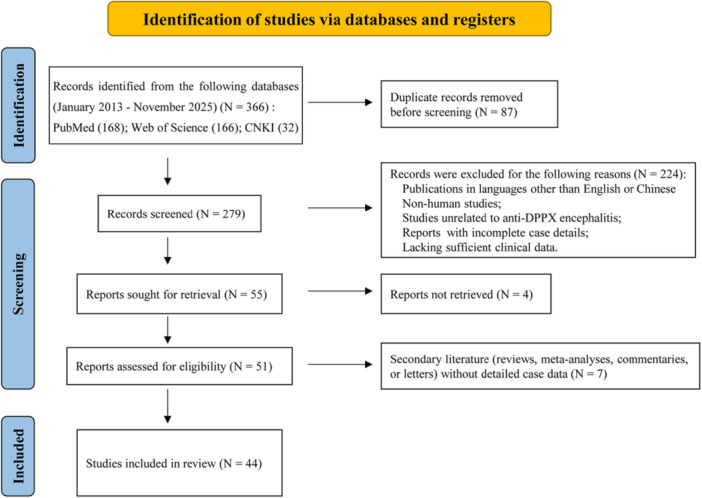
PRISMA flow diagram of study selection for the literature review.

This systematic review was conducted in accordance with established methodological and PRISMA guidelines. The review was not registered, and no protocol was prepared; therefore, no amendments are applicable. Due to the descriptive nature of the included studies (primarily case reports and case series), formal assessments of risk of bias, sensitivity analyses, reporting bias, and certainty of evidence were not applicable or feasible. We used the PRISMA 2020 reporting guideline [17] to draft this manuscript, and the PRISMA 2020 reporting checklist [18] was included in Supporting Information S1A and Figure 2 illustrates the study selection process using a PRISMA flow diagram. We used the CARE reporting guideline [19] to draft this manuscript, and the CARE reporting checklist [20] when editing, included in Supporting Information S2B.

### 3.2 Data Extraction and Management

Data extraction was independently performed by two trained neurologists using a predefined data collection form and standardized procedures to ensure data accuracy. For each included patient with DPPXE, the following information was collected: demographic characteristics (sex, age at onset, and region), clinical features, ancillary test results, treatment strategies, and follow‐up outcomes. All data were independently entered into a standardized electronic database (Microsoft Excel) by the two investigators and cross‐checked against the original reports. Discrepancies were resolved through re‐examination of the source data and adjudication by a third senior neurologist. Duplicate cases were identified by comparing institutional information, clinical characteristics, and follow‐up details; only the most comprehensive report was retained. Missing data were explicitly recorded without imputation. Upon completion of data curation, key variables were independently verified by a third investigator to ensure data integrity.

## 4 Statistical Analysis

Statistical analyses were performed using GraphPad Prism version 10.1.2. Descriptive statistics are presented as percentages for categorical variables and as medians with interquartile ranges (IQR) for continuous variables. Comparisons of continuous variables between groups were conducted using either the Student's *t*‐test or the Mann–Whitney *U*‐test based on data distribution. Categorical variables were compared using the chi‐square test, continuity correction test, or Fisher's exact test. A two‐sided *p*‐value < 0.05 was considered statistically significant.

## 5 Results

### 5.1 Summary of Demographic and Clinical Findings

A total of 125 patients were included in the analysis (comprising 124 previously reported cases and 1 patient from the present study). Demographic characteristics, clinical symptomatology, and summary of preclinical examinations are detailed in Table 1.

**Table 1:** Summary of clinical presentations of DPPXE from 2013 to 2025.

Clinical characteristics	*N* (%)	Clinical characteristics	*N* (%)
Gender (*n* = 125)		Gastrointestinal symptoms (*n* = 125)	
Male	78 (62.4%)	Gastrointestinal abnormal symptoms^a^	58 (46.4%)
Female	47 (37.6%)	Diarrhea	39 (31.2%)
Median age at onset (IQR)	52.0 (36.0, 61.0)	Nausea or vomiting	16 (12.8%)
Source of cases (*n* = 125)		Constipation	10 (8.0%)
China	62 (49.6%)	Abdominal pain	9 (7.2%)
United States	38 (30.4%)	Severe abdominal pain	2 (1.6%)
Spain	12 (9.6%)	Other symptoms (*n* = 125)	
Germany	7 (5.6%)	Weight loss	54 (43.2%)
Netherlands	2 (1.6%)	Headache	18 (14.4%)
Norway	1 (0.8%)	Associated tumor	14 (11.2%)
Slovenia	1 (0.8%)	Fever	13 (10.4%)
Canada	1 (0.8%)	Urinary abnormalities	9 (7.2%)
Australia	1 (0.8%)	Dyspnea	9 (7.2%)
Central nervous system symptoms (*n* = 125)		Dysphagia	4 (3.2%)
Cognitive impairment	82 (65.6%)	Pruritus	3 (2.4%)
Cerebellar or brainstem dysfunction symptoms	54 (43.2%)	EEG (*n* = 61)	
Sleep disorders	39 (31.2%)	Normal	31 (50.8%)
Memory decline	27 (21.6%)	Abnormal	30 (49.2%)
Dysarthria	7 (5.6%)	Brain MRI findings (*n* = 100)	
Attention deficits	5 (4.0%)	Normal	56 (56.0%)
Executive dysfunction	5 (4.0%)	Brain atrophy	14 (14.0%)
Disorientation	3 (2.4%)	White matter lesions	12 (12.0%)
Hyperexcitability symptoms		Cerebrospinal fluid examination (*n* = 103)	
CNS hyperexcitability	89 (71.2%)	Elevated protein quantification	50 (48.5%)
Seizures	32 (25.6%)	Normal	39 (37.9%)
Tremor	46 (36.8%)	Increased WBC count	32 (31.1%)
Myoclonus	34 (27.2%)	DPPX antibody tests (*n* = 90)	
Myotonia	17 (13.6%)	Serum	87 (96.7%)
Psychiatric symptoms (*n* = 125)		Cerebrospinal fluid	69 (76.7%)
Psychiatric abnormal symptoms	61 (48.8%)	DPPX antibody titer (IQR)	
Mood changes or depression	23 (18.4%)	Serum titer (*n* = 78)	1:100 (1:32, 1:320)
Hallucinations	19 (15.2%)	Cerebrospinal titer (*n* = 59)	1:32 (1:10, 1:160)
Excessive startle response	15 (12.0%)^a^		

Abbreviations: DPPXE, dipeptidyl‐peptidase‐like protein 6 antibody‐associated encephalitis; IQR, interquartile range; WBC, white blood cell.

^a^ Gastrointestinal abnormal symptoms were defined as the presence of any of the following: diarrhea, nausea/vomiting, constipation, or abdominal pain; as patients may experience more than one symptom, the sum of individual symptom counts may exceed the total number of patients with gastrointestinal abnormalities.

#### 5.1.1 Demographic Characteristics

This study included a total of 125 patients diagnosed with DPPXE from China, the United States, Germany, Spain, the Netherlands, Norway, and Slovenia. Among these, 62 cases were reported in China. The patients comprised 78 males (62.4%) and 47 females (37.6%); the age at onset predominantly involved middle‐aged individuals, with a median age of 52.0 (36.0, 61.0) years. The median age at onset was higher in males than in females (54.0 (37.0–62.0) years vs. 45.5 (35.0–60.0) years, *p* = 0.045). Cases were distributed across three continents, with China and the United States accounting for nearly 80% of the cohort. The remaining cases were sporadically reported from Europe and Oceania. These findings indicate a global sporadic distribution of the disease, with a higher concentration in the Asia‐Pacific and North American regions.

#### 5.1.2 Clinical Symptomatology

The clinical presentation is characterized by multisystem involvement, with neurocognitive impairment, increased neuronal excitability, and gastrointestinal dysfunction constituting the core symptom spectrum. The primary manifestations include CNS symptoms, heightened neuroexcitability, weight loss, and gastrointestinal disturbances. Among CNS symptoms, cognitive decline was the most prevalent, observed in 65.6% (82/125) of patients, encompassing deficits in memory, attention, executive function, and orientation. Cerebellar and brainstem involvement was noted in 43.2% (54/125), predominantly manifesting as ataxia, nystagmus, and respiratory failure. Sleep disturbances were reported in 31.2% (39/125) of cases. A majority of patients (71.2%, 89/125) exhibited signs of CNS hyperexcitability, characterized chiefly by seizures, tremors (both action and resting), and muscle rigidity. Behavioral and psychiatric abnormalities were present in 48.8% (61/125) of patients, predominantly including mood alterations or depression, hallucinations, and exaggerated startle responses; a minority exhibited personality changes and aggressive behavior.

The majority of patients (46.4%, 58/125) presented with prodromal symptoms indicative of gastrointestinal dysfunction; the most frequently reported symptoms were diarrhea, nausea and/or vomiting, constipation, and abdominal pain. Only a minority of patients experienced severe abdominal pain. Among patients with gastrointestinal dysfunction, 43.2% (54/125) demonstrated concomitant weight loss. In the subset of 40 patients with recorded weight loss measurements, the median reduction was 22.9 (13.6, 36.3) kg. The incidence of diarrhea was significantly higher in the weight‐loss group than in the non‐weight‐loss group (48.1% (26/54) vs. 19.7% (14/71)), indicating a significant association between weight loss and diarrhea (*p* = 0.001, OR = 3.79 (95% CI: 1.75–8.19)).

#### 5.1.3 Preclinical Examinations

Among the 125 patients with DPPXE reviewed in this study, EEG data were available for 61 cases, with a comparable distribution between normal and abnormal findings. In 90 patients who underwent both serum and CSF DPPX antibody testing, positivity rates were higher in serum (87/90, 96.7%) than in CSF (69/90, 76.7%). Brain MRI results were available for 100 patients; the majority demonstrated normal findings (56.0%, 56/100), whereas a minority exhibited cerebral atrophy and nonspecific white matter lesions. CSF analysis was conducted in 103 patients, with abnormal results predominantly characterized by elevated protein levels and increased white blood cell counts, while some patients had normal CSF parameters.

#### 5.1.4 Treatment and Outcomes

Among 125 patients diagnosed with DPPXE, 92.8% (116/125) received immunosuppressive therapy. The primary treatment modality was IVMP, administered in 75.0% (87/116) of cases, followed by IVIG in 47.4% (55/116), and plasma exchange (PLEX) in 33.6% (39/116). Combination immunotherapy regimens most commonly involved IVIG plus IVMP or IVMP plus PLEX. A minority of patients (28.4%, 33/116) also received adjunctive rituximab. Among 14 patients with concomitant malignancies, 71.4% (10/14) underwent tumor‐directed treatment, including chemotherapy, radiotherapy, and/or surgical resection. Among 109 patients with definitive follow‐up data, disease relapse was observed in 35 cases (32.1%, 35/109). Relapse was significantly more frequent in patients exhibiting tremors, exaggerated startle response, gastrointestinal symptoms (general gastrointestinal abnormalities and diarrhea), triptych, and CSF abnormalities (elevated protein concentration and increased WBC count) (Table 2).

**Table 2:** Clinical comparison of patients with relapse and non‐relapse DPPXE.

Clinical characteristics	Relapse (%)	Non‐relapse (%)	*p* value	OR (95% CI)
Sex (Male)	65.7 (23/35)	64.9 (48/74)	0.931^a^	1.04 (0.46–2.30)
Central nervous system symptoms				
Cognitive impairment	54.3 (19/35)	66.2 (49/74)	0.230^a^	0.61 (0.27–1.36)
Cerebellar or brainstem involvement	54.3 (19/35)	43.2 (32/74)	0.281^a^	1.56 (0.67–3.43)
Sleep disturbances	28.6 (10/35)	28.4 (21/74)	0.983^a^	1.01 (0.40–2.45)
Memory decline	25.7 (9/35)	16.2 (12/74)	0.240^a^	1.79 (0.70–4.83)
Dysarthria	2.9 (1/35)	8.1 (6/74)	0.532^b^	0.33 (0.03–2.22)
Attention deficits	2.9 (1/35)	4.1 (3/74)	0.814^b^	0.70 (0.05–4.83)
Executive dysfunction	0.0 (0/35)	2.7 (2/74)	> 0.999^c^	0.41 (0.02–8.76)^e^
Disorientation	0.0 (0/35)	2.7 (2/74)	> 0.999^c^	0.41 (0.02–8.76)^e^
Hyperexcitability symptoms				
CNS hyperexcitability	80.0 (28/35)	66.2 (49/74)	0.140^a^	2.04 (0.79–5.13)
Seizures	25.7 (9/35)	25.7 (19/74)	0.997^a^	1.00 (0.43–2.56)
Tremors	45.7 (16/35)	25.7 (19/74)	**0.036** a	2.44 (1.05–5.72)
Myoclonus	37.1 (13/35)	23.0 (17/74)	0.122^a^	1.98 (0.85–4.86)
Muscle rigidity	20.0 (7/35)	9.5 (7/74)	0.125^a^	2.39 (0.82–6.93)
Psychiatric symptoms				
Psychiatric abnormalities	48.6 (17/35)	45.9 (34/74)	0.798^a^	1.11 (0.51–2.39)
Mood changes or depression	17.1 (6/35)	17.6 (13/74)	0.829^a^	0.97 (0.33–2.87)
Hallucinations	20.0 (7/35)	13.5 (10/74)	0.384^a^	1.60 (0.60–4.40)
Exaggerated startle response	22.9 (8/35)	5.4 (4/74)	**0.007** a ^,^ **	5.19 (1.58–16.23)
Gastrointestinal symptoms				
General gastrointestinal abnormalities^f^	65.7 (23/35)	40.5 (30/74)	**0.014** a ^,^ *	2.81 (1.18–6.15)
Diarrhea	48.6 (17/35)	27.0 (20/74)	**0.027** a ^,^ *	2.55 (1.20–5.93)
Nausea or vomiting	14.3 (5/35)	12.2 (9/74)	0.757^a^	1.20 (0.42–3.98)
Constipation	8.6 (3/35)	5.4 (4/74)	0.833^b^	1.64 (0.39–6.38)
Abdominal pain	8.6 (3/35)	6.8 (5/74)	0.957^b^	1.29 (0.33–5.37)
Severe abdominal pain	0.0 (0/35)	1.4 (1/74)	0.700^c^	0.69 (0.03–17.60)^e^
Triptych	60.0 (21/35)	36.5 (27/74)	**0.021** a ^,^ *	2.61 (1.10–6.09)
Other symptoms				
Weight loss	48.6 (17/35)	36.5 (27/74)	0.230^a^	1.64 (0.75–3.61)
Headache	8.6 (3/35)	13.5 (10/74)	0.457^a^	0.60 (0.17–2.35)
Associated neoplasms	8.6 (3/35)	12.2 (9/74)	0.576^a^	0.68 (0.19–2.33)
Fever	8.6 (3/35)	9.5 (7/74)	0.837^b^	0.90 (0.24–3.62)
Urinary abnormalities	8.6 (3/35)	9.5 (7/74)	0.837^b^	0.90 (0.24–3.62)
Dyspnea	8.6 (3/35)	6.8 (5/74)	0.957^b^	1.29 (0.33–5.37)
Dysphagia	0.0 (0/35)	5.4 (4/74)	0.303^c^	0.22 (0.01–4.26)^e^
Pruritus	2.9 (1/35)	2.7 (2/74)	0.561^b^	1.06 (0.07–9.34)
DPPX antibody tests				
Serum	97.1 (34/35)	96.0 (71/74)	0.814^b^	1.44 (0.21–19.15)
Cerebrospinal fluid	55.6 (15/27)	63.3 (31/49)	0.511^a^	0.73 (0.28–1.94)
DPPX antibody titer^g^				
Serum	2.5 (1.5, 3.0) (*n* = 22)	2.0 (1.5, 3.1) (*n* = 57)	0.811^d^	NA
Cerebrospinal	1.9 (1.1, 2.2) (*n* = 15)	1.6 (0.6, 2.2) (*n* = 28)	0.717^d^	NA
EEG (Abnormal)	60.0 (9/15)	53.8 (21/39)	0.684^a^	1.29 (0.42–4.31)
Brain MRI findings				
Abnormal	54.8 (17/31)	50.8 (31/61)	0.715^a^	1.18 (0.49–2.75)
Cerebral atrophy	19.4 (6/31)	9.8 (6/61)	0.200^b^	2.20 (0.59–8.13)
White matter lesions	12.9 (4/31)	13.1 (8/61)	0.765^a^	0.98 (0.31–3.23)
Cerebrospinal fluid analysis				
Elevated protein concentration	64.5 (20/31)	39.0 (23/59)	**0.021** a ^,^ *	2.85 (1.14–7.15)
Increased WBC count	45.2 (14/31)	20.3 (12/59)	**0.014** a ^,^ *	3.23 (1.27–8.68)

*Note:* Bold *p* values denote statistical significance (*p* < 0.05).

Abbreviations: DPPXE, dipeptidyl‐peptidase‐like protein 6 antibody‐associated encephalitis; OR, odds ratio; WBC, white blood cell.

^a^ Pearson *χ* ^2^ test.

^b^ Continuity correction test.

^c^ Fisher's exact test.

^d^ Mann–Whitney *U*‐test.

^e^ OR and 95% confidence intervals (CI) were calculated using the Haldane‐Anscombe correction to account for zero cell counts.

^f^ Gastrointestinal abnormal symptoms were defined as the presence of any of the following: diarrhea, nausea/vomiting, constipation, or abdominal pain, as patients may experience more than one symptom, the sum of individual symptom counts may exceed the total number of patients with gastrointestinal abnormalities.

^g^ Values are expressed as −log_10_‐transformed antibody titers and presented as median (IQR).

* *p* < 0.05;

** *p* < 0.01.

### 5.2 Analysis of Differences in Clinical Manifestations

Differences exist in the sociodemographic characteristics, clinical manifestations, laboratory findings, and imaging results of patients with DPPXE in China compared to those reported in other countries. Specifically, Chinese patients with DPPXE demonstrated a lower prevalence of cerebellar or brainstem involvement, tremors, myoclonus, psychiatric symptoms (including psychiatric abnormalities, mood changes or depression, and exaggerated startle response), diarrhea, weight loss, DPPX antibody titer (serum and CSF), brain MRI imaging findings (cerebral atrophy), and CSF abnormalities (elevated protein concentration and increased WBC count). However, compared with patients from other countries, Chinese patients show a significantly higher prevalence of seizures, headache, fever, and abnormal brain MRI imaging findings. All of the above differences were statistically significant. Further detailed data are summarized in Table 3.

**Table 3:** Analysis of differences in clinical manifestations of DPPXE between China and other countries.

Clinical characteristics	Chinese	Other countries	*p* value	OR (95% CI)
Sex (Male)	59.7 (37/62)	65.1 (41/63)	0.533^a^	0.79 (0.40–1.69)
Central nervous system symptoms				
Cognitive impairment	66.1 (41/62)	65.1 (41/63)	0.902^a^	1.05 (0.51–2.15)
Cerebellar or brainstem involvement	30.6 (19/62)	55.6 (35/63)	**0.005** a ^,^ **	0.35 (0.17–0.74)
Sleep disturbances	25.8 (16/62)	36.5 (23/63)	0.197^a^	0.60 (0.29–1.28)
Memory decline	22.6 (14/62)	20.6 (13/63)	0.792^a^	1.12 (0.47–2.74)
Dysarthria	8.1 (5/62)	3.2 (2/63)	0.424^b^	2.68 (0.54–13.77)
Attention deficits	1.6 (1/62)	6.3 (4/63)	0.371^b^	0.24 (0.02–1.55)
Executive dysfunction	0.0 (0/62)	7.9 (5/63)	0.058^c^	0.09 (0.01–1.57)^e^
Disorientation	1.6 (1/62)	3.2 (2/63)	0.989^b^	0.50 (0.03–4.41)
Hyperexcitability symptoms				
CNS hyperexcitability	66.1 (41/62)	76.2 (48/63)	0.214^a^	0.61 (0.29–1.34)
Seizures	37.1 (23/62)	14.3 (9/63)	**0.004** a ^,^ **	3.54 (1.43–8.18)
Tremors	24.2 (15/62)	49.2 (31/63)	**0.004** a ^,^ **	0.33 (0.16–0.73)
Myoclonus	9.7 (6/62)	44.4 (28/63)	**< 0.0001** a ^,^ ****	0.13 (0.05–0.34)
Muscle rigidity	8.1 (5/62)	19.0 (12/63)	0.073^a^	0.37 (0.14–1.17)
Psychiatric symptoms				
Psychiatric abnormalities	38.7 (24/62)	58.7 (37/63)	**0.025** a ^,^ *	0.43 (0.22–0.88)
Mood changes or depression	6.5 (4/62)	30.2 (19/63)	**0.001** a ^,^ **	0.16 (0.06–0.47)
Hallucinations	9.7 (6/62)	20.6 (13/63)	0.088^a^	0.41 (0.15–1.15)
Exaggerated startle response	1.6 (1/62)	22.2 (14/63)	**0.001** b ^,^ **	0.06 (0.01–0.38)
Gastrointestinal symptoms				
General gastrointestinal abnormalities^f^	38.7 (24/62)	54.0 (34/63)	0.087^a^	0.54 (0.27–1.10)
Diarrhea	19.4 (12/62)	42.9 (27/63)	**0.005** a ^,^ **	0.32 (0.14–0.71)
Nausea or vomiting	12.9 (8/62)	12.7 (8/63)	0.973^a^	1.02 (0.34–3.01)
Constipation	6.5 (4/62)	9.5 (6/63)	0.762^b^	0.66 (0.20–2.46)
Abdominal pain	6.5 (4/62)	7.9 (5/63)	0.980^b^	0.80 (0.24–2.87)
Severe abdominal pain	3.2 (2/62)	0.0 (0/63)	0.244^c^	5.25 (0.25–111.44)^e^
Other symptoms				
Weight loss	16.1 (10/62)	69.8 (44/63)	**< 0.0001** a ^,^ ****	0.08 (0.03–0.20)
Headache	21.0 (13/62)	7.9 (5/63)	**0.038** a ^,^ *	3.08 (1.02–8.19)
Associated neoplasms	9.7 (6/62)	12.7 (8/63)	0.592^a^	0.74 (0.23–2.40)
Fever	21.0 (13/62)	0.0 (0/63)	**< 0.0001** c ^,^ ****	34.60 (2.01–593.00)^e^
Urinary abnormalities	3.2 (2/62)	11.1 (7/63)	0.174^b^	0.27 (0.05–1.30)
Dyspnea	3.2 (2/62)	11.1 (7/63)	0.174^b^	0.27 (0.05–1.30)
Dysphagia	1.6 (1/62)	4.8 (3/63)	0.623^b^	0.33 (0.02–2.27)
Pruritus	0.0 (0/62)	4.8 (3/63)	0.244^c^	0.14 (0.01–2.72)^e^
DPPX antibody tests				
Serum	98.4 (61/62)	95.2 (60/63)	0.623^b^	3.05 (0.44–40.18)
Cerebrospinal fluid	58.5 (31/53)	60.9 (28/46)	0.810^a^	0.91 (0.42–2.01)
DPPX antibody titer^g^				
Serum	1.5 (1.0, 2.0) (*n* = 42)	3.1 (2.7, 3.9) (*n* = 36)	**< 0.0001** d ^,^ ****	NA
Cerebrospinal fluid	1.0 (0.0, 1.5) (*n* = 31)	2.2 (1.6, 2.5) (*n* = 28)	**< 0.0001** d ^,^ ****	NA
EEG (Abnormal)	63.9 (23/36)	44.0 (11/25)	0.124^a^	2.25 (0.75–6.35)
Brain MRI findings				
Abnormal	53.2 (33/62)	28.9 (11/38)	**0.018** a ^,^ *	2.79 (1.21–6.50)
Cerebral atrophy	6.5 (4/62)	26.3 (10/38)	**0.013** b ^,^ *	0.19 (0.06–0.67)
White matter lesions	12.9 (8/62)	10.5 (4/38)	0.970^a^	1.26 (0.39–3.99)
Cerebrospinal fluid analysis				
Elevated protein concentration	40.3 (25/62)	61.0 (25/41)	**0.040** a ^,^ *	0.43 (0.19–0.98)
Increased WBC count	12.9 (8/62)	58.5 (24/41)	**< 0.0001** a ^,^ ****	0.10 (0.04–0.28)

*Note:* Bold *p* values denote statistical significance (*p* < 0.05).

Abbreviations: DPPXE, dipeptidyl‐peptidase‐like protein 6 antibody‐associated encephalitis; OR, odds ratio; WBC, white blood cell.

^a^ Pearson *χ* ^2^ test.

^b^ Continuity correction test.

^c^ Fisher's exact test.

^d^ Mann–Whitney *U*‐test.

^e^ OR and 95% confidence intervals (CI) were calculated using the Haldane‐Anscombe correction to account for zero cell counts.

^f^ Gastrointestinal abnormal symptoms were defined as the presence of any of the following: diarrhea, nausea/vomiting, constipation, or abdominal pain, as patients may experience more than one symptom, the sum of individual symptom counts may exceed the total number of patients with gastrointestinal abnormalities.

^g^ Values are expressed as −log_10_‐transformed antibody titers and presented as median (IQR).

* *p* < 0.05;

** *p* < 0.01;

**** *p* < 0.0001.

## 6 Discussion

DPPXE is a rare autoimmune encephalitis with an incompletely understood etiology, potentially associated with infection, immune dysregulation, or underlying neoplasms [14]. DPPX is widely expressed in both the CNS (including the hippocampus, cerebellum, brainstem, and striatum) and the peripheral nervous system (notably within the enteric nervous system) [2, 3, 4]. The production of anti‐DPPX antibodies can result in multisystem dysfunction, manifesting with considerable clinical heterogeneity. As previously described, symptoms in DPPXE encompass the characteristic “triptych” as well as a range of nonspecific manifestations, reflecting the broad distribution of DPPX throughout the human body [2, 3, 6, 9, 14]. DPPXE typically presents in its early stages with neuropsychiatric symptoms such as behavioral abnormalities, cognitive impairment, seizures, sleep disturbances, or refractory gastrointestinal symptoms, including persistent diarrhea; however, these initial manifestations are often nonspecific and frequently misdiagnosed as primary psychiatric or gastrointestinal disorders [13, 21, 22, 23]. Limited awareness among clinicians regarding this rare antibody‐mediated condition, combined with the absence of comprehensive early‐stage neural antibody testing, may contribute to frequent misdiagnosis or missed diagnosis during the initial disease course. Many patients first seek medical attention in psychiatric or gastroenterology departments. Furthermore, neuroimaging and EEG findings are generally nonspecific, further complicating the clinical diagnosis [24]. These factors render the early diagnosis of DPPXE highly challenging. Previous studies have reported diagnostic delays ranging from several months to years in cases of anti‐DPPX antibody encephalitis [13, 14, 21, 22, 23, 25]. In our case, there was a prolonged interval between symptom onset and definitive diagnosis, primarily characterized by refractory gastrointestinal symptoms, notably severe abdominal pain, which are frequently misattributed to primary gastrointestinal disorders. These atypical and heterogeneous initial presentations often obscure the underlying diagnosis of DPPXE, resulting in diagnostic delay of approximately 3 years. Therefore, a comprehensive understanding of these heterogeneous clinical manifestations is crucial for enhancing neurologists' recognition of the disease, thereby facilitating earlier diagnosis and timely intervention.

Specifically, the case reported in this study exhibited rare clinical features, severe abdominal pain manifested in two distinct patterns: episodes temporally associated with seizures and isolated episodes without accompanying epileptic features. Both during the prior disease course and the current hospitalization, severe abdominal pain occurred almost exclusively as isolated episodes. Colonoscopic examination revealed only chronic colitis, which was unlikely to fully account for the severity of the abdominal pain observed in this patient. Other potential causes of abdominal pain, including gastrointestinal disorders and hereditary diseases (such as acute intermittent porphyria), were systematically excluded based on the patient's medical history and comprehensive diagnostic workup. The positive result of serum anti‐DPPX antibodies and the absence of epileptiform discharges on video‐EEG during episodes of isolated abdominal pain strongly suggested that this symptom was associated with the anti‐DPPX immune response. A repeat serum test yielding positive results further substantiated the diagnostic reliability. The epileptic manifestations in our patient aligned with the typical clinical features of anti‐DPPXE reported in previous studies, whereas the prominent presentation of severe abdominal pain was rarely described in the literature. We have noted that a recent systematic review by Li et al. [23] reported the viewpoints of “Approximately 44.2% (41/104) of patients presented with a triad of gastrointestinal symptoms (predominately diarrhea)” and “Gastrointestinal abnormalities (with diarrhea and abdominal pain being the most common) were reported in 54.8% (57/104) of patients with DPPXE.” Based on findings from the present study and prior reports of DPPXE, while diarrhea is the predominant gastrointestinal symptom, abdominal pain is also a common associated feature. However, isolated, severe, paroxysmal abdominal pain, as seen in this case, appears to be a rare and noteworthy presentation of DPPXE. For instance, previous reports have clearly documented nausea in 16 patients [6, 11, 14, 16, 26, 27, 28, 29, 30], whereas abdominal pain was reported in only 8 cases [2, 6, 14, 15, 16]. A possible explanation is that previous case reports often described “gastrointestinal symptoms” in vague terms, leading to potential information bias. Further retrospective review of the clinical data from these 8 patients revealed that although 7 patients initially presented with abdominal pain, this symptom was not the predominant factor affecting their quality of life and the severity of abdominal pain in these cases was generally less intense than that observed in the present case [2, 10, 14, 15, 16], one patient from Slovenia even experienced spontaneous resolution of abdominal pain before hospitalization [2]. To our knowledge, only one case has been previously reported: involving a 72‐year‐old male patient described by Zhou et al. [10], who presented with severe abdominal pain. Collectively, these findings indicate that DPPXE patients exhibiting severe abdominal pain as a predominant clinical manifestation remain exceedingly rare.

However, the underlying mechanism of abdominal pain in patients with DPPXE remains unclear. Previous studies have demonstrated that DPPX is highly expressed in enteric neurons in addition to the CNS, and autoantibodies targeting the DPPX/Kv4.2 channel complex in the gastrointestinal tract exert modulatory effects, leading to hyperexcitability of enteric neurons, which may contribute to abdominal pain and other gastrointestinal symptoms [2, 31]. Zhou et al. [10] provided a similar explanation for the severe abdominal pain observed in their cases, supporting this pathogenic mechanism. However, Boronat et al. [2] hypothesized that the gastrointestinal symptoms associated with DPPXE may not be solely attributable to local neuronal dysfunction but could involve a molecular mimicry mechanism linked to an unidentified infectious agent, analogous to the pathogenesis of anti‐GM1 antibody production induced by Campylobacter jejuni infection in Guillain‐Barré syndrome. Whether the aforementioned mechanisms are directly involved in the pathogenesis of abdominal pain remains unclear and requires further mechanistic investigation for validation. Recent studies have proposed the concept of the “gut–brain axis” as a bidirectional communication pathway between the brain and the gastrointestinal tract, involving complex interactions among neural, immune, endocrine pathways, and the gut microbiota [32, 33]. Notably, the unique clinical presentation in our case (epileptic seizures accompanied by severe abdominal pain) suggests a potential link between central and peripheral systems; it is hypothesized that, in addition to the previously discussed mechanisms, the severe abdominal pain experienced by this patient may also result from activation of the gut–brain axis, mediated by inflammatory factors or neurotransmitters released during abnormal CNS activity. This possibility warrants further investigation to elucidate the underlying pathological and physiological basis in future studies.

According to the *2016 Lancet Neurology* diagnostic approach proposed by Graus et al. [9], autoimmune encephalitis should initially be classified using a general, syndrome‐based framework that relies on the clinical course, core encephalitic features, supportive paraclinical findings, and the reasonable exclusion of alternative etiologies. Our patient fulfilled these criteria by presenting with encephalitic syndrome characterized by recurrent seizures and severe abdominal pain. Extensive diagnostic work‑up, including brain MRI, EEG, chest and abdominal CT, and comprehensive serum and CSF investigations, reasonably excluded alternative causes such as neoplasms, abdominal epilepsy, primary gastrointestinal disorders, and hereditary metabolic diseases. Of particular importance, the reproducible detection of serum anti‑DPPX antibodies further supported an autoimmune etiology. In current practice, the diagnosis of DPPXE is challenging because clinical manifestations, neuroimaging, and electrophysiological features are often non‑specific and rarely pathognomonic. Consequently, definitive diagnosis primarily relies on the detection of anti‐DPPX antibodies in serum or CSF, in conjunction with clinical presentation and the exclusion of alternative etiologies [2, 6, 9]. The diagnosis of our case was supported by two independent positive serum DPPX antibody results (both with a titer of 1:10), whereas CSF was tested once with negative results. Although a negative CSF result and low‐titer serum positivity were each insufficient to establish the diagnosis, two concordant positive serum tests increase diagnostic reliability. CSF routine analysis, MRI, and EEG findings in this case were nonspecific, consistent with previous reports [12, 14, 16, 24, 34]. In the differential diagnosis, abdominal epilepsy was systematically evaluated: continuous 48‐h EEG monitoring captured two discrete episodes of abdominal pain without accompanying epileptic features, and no epileptiform activity was detected during these events. These findings argue against an epileptic etiology and further support the diagnosis of DPPX antibody–associated disease.

Our data analysis indicates that, among patients diagnosed with anti‐DPPX encephalitis, the detection rate of DPPX antibodies is higher in serum (96.7%) than in CSF (76.7%), which is consistent with the findings reported by Miao et al. [21]. Furthermore, previous studies have also consistently demonstrated that serum anti‐DPPX antibody titers are generally higher than those in CSF, exhibiting a markedly greater magnitude [14, 35], which is consistent with our analysis. Based on the findings of existing studies, the variability in detection rates of anti‐DPPX antibodies may be attributed to their sites of production and distribution: on the one hand, anti‐DPPX antibodies are predominantly generated in peripheral immune organs such as the spleen and lymph nodes, with a smaller proportion synthesized within the CNS's intrathecal compartment [6]; on the other hand, antibodies produced by peripheral immune responses circulate systemically via the bloodstream, the blood‐brain barrier, serving as a highly selective physiological barrier, substantially restricts the passage of anti‐DPPX antibodies from the peripheral circulation into the CSF, this restriction consequently results in relatively low concentrations of antibodies and reduced detection rates in the CSF. It is important to note that there is currently no definitive evidence establishing the sensitivity and specificity of DPPX antibody detection in serum or CSF, therefore, the diagnosis of DPPXE should not be excluded solely on the basis of a negative result in either serum or CSF, when results are negative in one biological fluid, repeated testing of the other should be performed to minimize the risk of missed diagnosis [36].

Undoubtedly, the presence of DPPX antibodies is one of the key criteria for the diagnosis of DPPXE; however, the relationship between antibody titer and clinical diagnosis remains uncertain [9, 23]. Several previous reports have demonstrated that even low titers of DPPX antibodies (e.g., 1:10 to 1:32) may support a diagnosis of DPPXE when the patient's clinical features are consistent with autoimmune encephalitis and alternative etiologies have been excluded [11, 21, 24]. This suggests that low‐titer antibodies can retain significant diagnostic value when interpreted in the context of comprehensive clinical evaluation. Moreover, there is currently no evidence supporting a consistent association between antibody titer levels and either disease severity or risk of relapse. For instance, some patients with low titers have exhibited marked neuropsychiatric symptoms and experienced favorable outcomes following immunotherapy [14, 21, 23]. In other forms of antibody‐mediated autoimmune encephalitis, antibody titers exhibit a dynamic course characterized by a decline over time, particularly following immunotherapy. Longitudinal studies of anti‐NMDAR, LGI1, and CASPR2 encephalitis show that serum and CSF antibody titers decrease substantially from disease onset, with some patients becoming seronegative after clinical remission; however, titers may rise again in a subset of patients in association with relapse [37, 38, 39]. The patient described in our study had a disease duration exceeding 3 years; the repeat testing during the current acute episode revealed a low serum antibody titer, which may be attributable to the prolonged, chronically progressive disease course. For anti‐DPPX antibodies, available evidence regarding longitudinal titer dynamics remains limited. Most existing cohort studies report only baseline serum and/or CSF titers at diagnosis, with a lack of systematic longitudinal follow‐up data. Further studies are urgently needed to elucidate the temporal dynamics of anti‐DPPX antibody levels. Importantly, in antibody‐mediated autoimmune encephalitis (including DPPXE), low serum or CSF antibody titers in the later stages of a chronic disease course are not sufficient to exclude the diagnosis. Instead, antibody results must be interpreted within the broader clinical context, including disease activity, prior immunotherapy, and the timing of sample collection. In our study, the reported case demonstrated a repeating serum DPPX antibody titer of 1:10 and was ultimately diagnosed with DPPXE based on a comprehensive assessment of clinical presentations, antibody testing, and exclusion of other potential etiologies.

In terms of treatment, immunotherapy remains the cornerstone of treatment for autoimmune encephalitis and has been demonstrated to be highly effective in alleviating symptoms and improving prognosis [28, 40]. Current therapeutic strategies commonly include glucocorticoids, IVIG, PLEX, and more targeted immunomodulatory agents. Glucocorticoids are typically employed as first‐line therapy to achieve rapid suppression of the inflammatory response. IVIG and PLEX contribute to immune system modulation by supplying immunoregulatory factors and removing pathogenic antibodies, respectively [2, 6, 25, 28, 41]. The present patient demonstrated significant clinical improvement in both epileptic symptoms and severe abdominal pain following treatment with IVMP and immunoglobulin, indicating the efficacy of immunotherapy in DPPXE. This therapeutic response is consistent with previously reported treatment outcomes [2, 6, 16, 23, 25, 28, 41, 42, 43, 44, 45]. Furthermore, the post‐discharge therapeutic course and follow‐up outcomes provide additional support for the attribution of severe abdominal pain to DPPXE in our case. Notably, the majority of patients experience symptomatic improvement or even complete recovery following treatment; however, a considerable proportion still develop disease relapse [13, 24]. Our retrospective analysis in the present study demonstrated that recurrence was significantly more common among patients presenting with tremors, exaggerated startle response, gastrointestinal symptoms (general gastrointestinal abnormalities and diarrhea), triptych, and CSF abnormalities (elevated protein concentration and increased WBC count); these differences were statistically significant. Nevertheless, this is an associative analysis, indicating that the aforementioned factors may just represent potential risk indicators for recurrence, rather than establishing a definitive causal relationship.

There may be some differences in the clinical characteristics of DPPXE patients between China and other countries. For instance, the study by Xiao et al. [24] has demonstrated that the proportion of Chinese patients exhibiting encephalitis‐specific abnormalities on brain MRI was higher than that reported in other populations. Our study further suggested that Chinese patients may differ from those in other countries in the incidence of several clinical and paraclinical features. Chinese patients with DPPXE exhibited a lower prevalence of cerebellar or brainstem involvement, tremors, myoclonus, psychiatric symptoms (including psychiatric abnormalities, mood changes or depression, and exaggerated startle response), diarrhea, weight loss, DPPX antibody titer (serum and CSF), brain MRI imaging findings (cerebral atrophy), and CSF abnormalities (elevated protein concentration and increased WBC count). In contrast, compared with patients from other countries, Chinese patients show significantly higher prevalence of seizures, headache, fever, and abnormal brain MRI imaging findings. However, the reasons underlying these observed differences remain unclear, and several potential factors may account for the variations observed between Chinese and other populations: at the biological level, differences in genetic background and ethnicity‐related immune responses may contribute to variations in autoimmune activity as well as to the observed differences in serum and CSF antibody titers across populations, which may further be influenced by the timing of sample collection and assay methodologies. Clinically, variability in physician awareness, diagnostic experience, and evaluation strategies across centers may contribute to differences in clinical recognition and the reported frequency of neuroimaging and CSF abnormalities. From a publication perspective, a reporting bias cannot be excluded, as more severe or clinically typical cases are more likely to appear in the literature. Finally, sociocultural and environmental factors, including dietary patterns, gut microbiota composition, and healthcare‐seeking behaviors, may further modulate disease manifestation. However, the above considerations represent possible explanations or hypotheses rather than definitive conclusions. Future large‐scale, prospective, multicenter, and international collaborative studies are warranted to validate these observations, elucidate the underlying mechanisms, and provide a more comprehensive understanding of the clinical spectrum of this disease.

## 7 Limitations

This study has several inherent limitations related to its retrospective design. First, the reviewed cases were identified from previously documented medical records, in which clinical data were occasionally incomplete or inconsistently recorded, thereby limiting a comprehensive evaluation of disease course, treatment response, and outcomes. Second, restricting inclusion to publications in English and Chinese may have introduced language and publication bias. Third, given the rarity of DPPXE, the overall sample size was relatively small, which may have reduced statistical power and limited the generalizability of our findings. Fourth, variability in diagnostic criteria, ancillary investigations, and treatment strategies across centers likely introduced heterogeneity, potentially affecting the consistency and comparability of the results. Finally, the absence of long‐term follow‐up data precluded a systematic assessment of long‐term outcomes. Future multicenter, large‐scale prospective studies are warranted to validate these findings, further characterize the clinical spectrum of DPPXE, and optimize evidence‐based therapeutic strategies.

## 8 Conclusion

DPPXE is an exceedingly rare autoimmune neurological disorder characterized by marked clinical heterogeneity. In this study, we report a case of a Chinese patient presenting predominantly with severe abdominal pain, thereby expanding the clinical spectrum of DPPXE. Additionally, we performed a systematic review and comprehensive analysis of all published DPPXE cases to date, summarizing their clinical manifestations, relapse patterns, and therapeutic responses. Furthermore, by comparing the clinical features of Chinese patients with those reported internationally, we preliminarily identified potential differences in clinical presentation and disease progression between these populations. Collectively, our findings provide up‐to‐date and comprehensive insights into the pathophysiology and clinical course of this rare disorder from multiple perspectives.

## Author Contributions

**Difang Shi:** data curation, formal analysis, writing – original draft, writing – review and editing, software. **Haohao Wu:** data curation, formal analysis, investigation, methodology. **Baogang Huang:** conceptualization, writing – review and editing. **Yan Zheng:** conceptualization, writing – review and editing. **Jia Liu:** writing – review and editing, conceptualization, validation, resources. **Jianjian Bao:** conceptualization, writing – review and editing. **Fengming Xu:** data curation, writing – review and editing, writing – original draft, visualization. **Kang Du:** data curation, writing – original draft, writing – review and editing, supervision, funding acquisition, project administration. All authors contributed to the article and approved the submitted version.

## Ethics Statement

The studies involving human participants were conducted in accordance with the Helsinki Declaration and approved by the Ethical Committee of Qujing First People's Hospital (Approval No. 202300801).

## Consent

The written informed consent for publication of identifying images or other personal or clinical details was obtained from the participant in this study.

## Conflicts of Interest

The authors declare no conflicts of interest.

## AI Statement

The authors declare that no artificial intelligence (AI) tools were used in the writing or preparation of this manuscript.

## Supporting information

Supporting File 1

Supporting File 2

## Data Availability

The original contributions presented in the study are included in the article/Supporting material. Further inquiries can be directed to the corresponding authors.
